# CTHRC1 is a prognostic biomarker correlated with immune infiltration in head and neck squamous cell carcinoma

**DOI:** 10.1186/s12903-024-04525-x

**Published:** 2024-06-27

**Authors:** Zhichao Zhang, Xusheng Ren, Yiling Wang, Ping Liu, Peng Lin, Shumei Jin, Chao Xu

**Affiliations:** 1 Department of Oral and Maxillofacial Surgery, The Second Hospital of Shandong University, 247 Beiyuan Street, Jinan, Shandong China; 2 Department of Orthodontics, Jinan Stomatological Hospital, 101 Jingliu Road, Jinan, Shandong China

**Keywords:** CTHRC1, Bioinformatics, Tumor microenvironment, Immune infiltration, Biomarker, HNSCC

## Abstract

**Background:**

Head and neck squamous cell carcinoma (HNSCC) is the sixth most common malignancy worldwide, characterized by high morbidity, high mortality, and poor prognosis. Collagen triple helix repeat containing 1 (CTHRC1) has been shown to be highly expressed in various cancers. However, its biological functions, potential role as a biomarker, and its relationship with immune infiltrates in HNSCC remain unclear. Our principal objective was to analyze CTHRC1 expression, its prognostic implications, biological functions, and its effects on the immune system in HNSCC patients using bioinformatics analysis.

**Methods:**

The expression matrix was obtained from The Cancer Genome Atlas (TCGA) and Gene Expression Omnibus (GEO). CTHRC1 expression in HNSCC was analyzed between tumor and adjacent normal tissues, different stages were compared, and its impact on clinical prognosis was assessed using Kaplan-Meier analysis. Gene Ontology (GO), Kyoto Encyclopedia of Genes and Genomes (KEGG), and Gene Set Variation Analysis (GSVA) were employed for enrichment analysis. The Search Tool for the Retrieval of Interacting Genes database (STRING) was used to analyze protein-protein interactions. Pearson correlation tests were used to investigate the association between CTHRC1 expression and immune checkpoints. The correlation between CTHRC1 and immune infiltration was investigated using CIBERSORT, TIMER, and ESTIMATE.

**Results:**

Compared to adjacent normal tissues, CTHRC1 was found to be highly overexpressed in tumors. Increased expression of CTHRC1 was more evident in the advanced stage of HNSCC and predicted a poor prognosis. Most genes related to CTHRC1 in HNSCC were enriched in physiological functions of Extracellular matrix(ECM) and tumor. Furthermore, several immune checkpoints, such as TNFSF4 and CD276 have been shown to be associated with CTHRC1 expression. Notably, the level of CTHRC1 expression correlated significantly with immune infiltration levels, particularly activated macrophages in HNSCC.

**Conclusions:**

High expression of CTHRC1 predicts poor prognosis and is associated with immune infiltration in HNSCC, confirming its utility as a tumor marker for HNSCC.

**Trial registration:**

Not applicable. All data are from public databases and do not contain any clinical trials.

## Introduction

Head and neck squamous cell carcinoma, originates from various subsites of the upper aerodigestive tract, including the oral cavity, sinonasal cavity, pharynx, and larynx [1, 2]. Smoking, alcohol consumption, exposure to environmental pollutants, and infection with HPV and EB viruses are the primary risk factors for HNSCC [3–5]. According to the latest global cancer statistics, in 2022, HNSCC account for 1,026,891 new cases and 458,107 deaths, ranking sixth in both incidence and mortality rates [6]. Comprehensive sequential therapy based on surgery has been considered the optimal treatment for HNSCC [7]. Despite ongoing efforts in risk stratification and treatment for patients with HNSCC, the response to treatment and survival rates for those with advanced HNSCC remain considerably lower compared to the early stage due to its high heterogeneity and invasiveness [8]. Given that over 50% of HNSCC patients are diagnosed in the middle and late stages of cancer progression, there is an urgent need for early detection and new treatment strategies for HNSCC to enhance disease control and prevention.

In recent years, high-throughput sequencing technology has rapidly evolved, generating large scale biological data and proving to be an effective tool for discovering promising cancer biomarkers [9, 10]. Through bioinformatic analysis and traditional method such as immunohistochemical staining, various gene markers for cancer were identified, including AFP, EGFR, P21 and CD79 [11–14]. Identifying genes that significantly correlate with the progression and prognosis in HNSCC patients may be applicable for establishing a robust model for HNSCC diagnosis and treatment. CTHRC1 is an extracellular matrix (ECM) protein encoded by a gene on human chromosome 8q22.3 [15]. CTHRC1 has been demonstrated to play a pivotal role in various physiological and pathological processes, including vascular remodeling, cell migration, lymph node metabolism, and mineralization of bone [16, 17]. Recent research has revealed that the expression of CTHRC1 is positively correlated with tumor stage and prognosis in various tumors [18]. For example, Lai et al. demonstrated that CTHRC1 promotes cell proliferation and metastasis, indicating a poor prognosis in breast cancer [19]. Additionally, Zhao et al. proposed that CTHRC1 might be a promising novel target for immunotherapy and angiogenesis in gastric cancer [20]. Similarly, other cancers, including non-small cell lung cancer, colon adenocarcinoma, and oral cancer, have also demonstrated a close association with CTHRC1 [21–23]. Although previous studies have highlighted the high expression of CTHRC1 in HNSCC patients [23], the predictive accuracy of CTHRC1 as a biomarker has not been confirmed due to a lack of samples and rigorous bioinformatic analysis.

Over the past decade, immunotherapy has achieved significant success in various cancers [24, 25]. However, a multitude of studies have revealed that a considerable number of HNSCC patients exhibit insensitivity to current immunotherapies, indicating an immunosuppressed state [26–28]. To our knowledge, the tumor microenvironment (TME) of HNSCC consists of a complex system of immune cells, playing a vital role in tumor growth, metastasis, invasiveness and escape, and may be a potential cause of the immunosuppressed state [29, 30]. Tumor associated macrophages (TAMs) in HNSCC often exhibit a M2 phenotype associated with poor prognosis, while CD8 + cytotoxic T cells and NK cells generally correlate with better prognosis [31, 32]. Dendritic cells (DCs) play a crucial role in antigen presentation, but dysfunctional or immature DCs in the TME can lead to ineffective anti-tumor immunity [33]. Studies have demonstrated that various cancer markers are linked to immune infiltration in TME. High levels of PD-L1 expression are generally correspond with decreased infiltration of tumor infiltrating lymphocytes, especially CD8^+^ T cells [34], while TP53 mutation is associated with reduced CD8^+^ T cell infiltration and increased regulatory T cell infiltration [35]. Recent bioinformatics analyses have also shown that a variety of tumor markers are correlated with tumor immune infiltration in HNSCC, such as PER3, IDO1, MYL1 and HRPT1 [36–38]. Exploring the relationship between tumor markers and immune infiltration is crucial for understanding tumor immune status, predicting and improving the efficacy of immunotherapy, and identifying new targets for treatment.

In this study, we systematically analyzed the expression status and prognostic value of CTHRC1 in head and neck squamous cell carcinoma using data from the TCGA and GEO database. GO, KEGG, and GSVA were employed to uncover the primary biological functions of genes associated with CTHRC1 expression. A protein–protein interaction network was constructed to explore the interactions among relevant proteins. Analysis of tumor-infiltrating immune cells was conducted to unveil the relationship between the TME and CTHRC1 expression, aiming to identify a novel gene marker for the early diagnosis and treatment of HNSCC.

## Methods

### Database

A total of 566 samples of head and neck squamous cell carcinoma with gene expression data were obtained from TCGA (https://tcga-data.nci.nih.gov/tcga/), comprising 522 cancer tissues of HNSCC and 44 adjacent cancer tissues. The “easyTCGA” R package was utilized for downloading and processing the data, while “dplyr” was employed for data standardization in preparation for subsequent analyses. Clinical information, including gender, age, clinical stage, pathologic stage, site or organ of origin, survival status, and survival duration in months, was also downloaded. The Tumor Immune Estimation Resource (TIMER), Gene Expression Profiling Interactive Analysis (GEPIA) and the Human Protein Atlas database (HPA) were also utilized in this study. GSE30784 and GSE41613 from GEO (https://www.ncbi.nlm.nih.gov/geo) were used to validate the results. All the data were accessible following the provided guidelines, with no restrictions or limitations.

### Analysis of gene expression between the tumor and normal tissue

The TIMER 2.0 database (https://cistrome.shinyapps.io/timer) was employed for pan-cancer analysis, and the results for HNSCC were subsequently validated using the GEPIA database (http://gepia.cancer-pku.cn). The following analysis was performed using R (version 4.3.2). The mRNA expression matrices of 522 tumor tissues and 44 normal samples were downloaded from TCGA and normalized. The “limma” package was employed to analyze differentially expressed genes (DEGs) between normal and tumor tissues. Subsequently, a volcano plot was generated using the “ggplot2” and “volcano” R packages. Analysis thresholds were set with an absolute fold change greater than 1 and a *P* value less than 0.05. Genes exhibiting significantly increased expression are depicted as red dots on the right side of the plot, while genes with significantly decreased expression are represented as blue dots on the left side. Since the results of GEPIA and TIMER were based on TCGA database, GSE30784 from GEO database was used to validate the results of the above studies. Immunohistochemical images from the HPA database (https://www.proteinatlas.org) were utilized to qualitatively assess CTHRC1 expression in both tumor and normal tissues at the histological level.

### CTHRC1 expression between different clinical phenotypes

The normalized expression matrix was employed to analyze differences in expression between phenotypes. Data were statistically analyzed using IBM SPSS Statistics (version 25.0), and homogeneity of variance was verified. The t-test or one-way analysis of variance was employed to calculate differences between subgroups. *P* values less than 0.05 were considered indicative of a significant difference. Box plots were generated using the “ggplot2” package.

### Prognostic analysis

Patient survival data were obtained from TCGA and GSE41613. Overall survival (OS) was analyzed using the Kaplan-Meier method. The grouping into high-level and low-level mRNA expression was determined using maximally selected rank statistics. A *P* value < 0.05 was considered indicative of statistical significance.

### GO and KEGG analysis

Pearson correlation analysis was performed using R (version 4.3.2) to uncover the correlation between CTHRC1 expression and other genes. Subsequently, the top 500 relevant genes were selected and imported into the database for Annotation, Visualization, and Integrated Discovery (DAVID) (https://david.ncifcrf.gov). The DAVID is a database utilized for GO analysis, including biological process (BP), cellular component (CC), and molecular function (MF). KEGG analysis was also conducted using these genes. The results of the analysis were visualized using the “ggplot2” package.

### GSVA analysis

The associated gene sets were obtained from “AmiGO2” (https://amigo.geneontology.org/). The functional enrichment score for each sample was computed using the “GSVA” R package with default parameters. The results were visualized through a heatmap, and pearson correlation analysis was employed to assess the correlation between CTHRC1 and the specified gene sets.

### Construction of protein–protein interaction network

To investigate the interaction among related proteins, we constructed a protein-protein interaction network using the STRING database (https://string-db.org/). A minimum required interaction score of 0.4 was set. The protein-protein interaction (PPI) network was visualized using Cytoscape software. Subsequently, GO and KEGG analyses were performed based on the genes encoding these proteins, following the previously mentioned method.

### Immune checkpoint testing

A total of 79 immune checkpoints were retrieved from a previous study [39]. Pearson correlation analysis was employed to examine the correlations between CTHRC1 expression and these checkpoints. A *P* value of less than 0.05 was considered statistically significant. Subsequently, a doughnut diagram was generated using the provided R package in the default parameters of the R environment to visualize the results.

### Tumor infiltrating immune cell analysis

Tumor infiltrating immune cell analysis was performed using Cell-type Identification By Estimating Relative Subsets Of RNA Transcripts (CIBERSORT) to characterize the immune features of HNSCC. Immune scores were calculated using the Estimation of Stromal and Immune Cells in Malignant Tumor Tissues using Expression Data (ESTIMATE) algorithm to assess the presence of immune cells. Also, the correlations between CTHRC1 expression levels and the level of immune infiltration were estimated using TIMER, a comprehensive resource for analyzing immune infiltrates in gene expression profiles.

## Results

### CTHRC1 expression in normal tissues and tumors

**Fig. 1:**
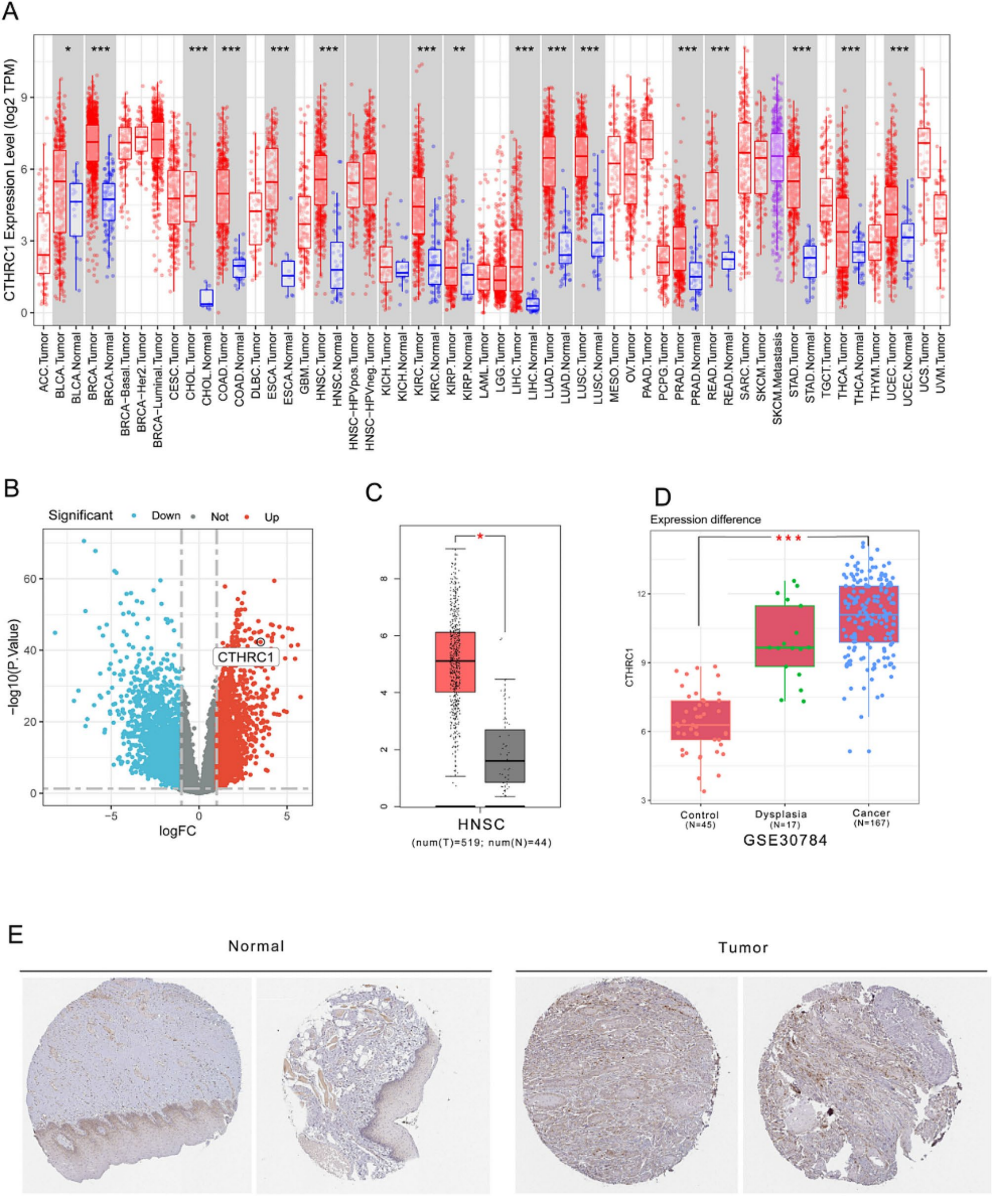
Expression analysis of CTHRC1 in different databases. **A** Expression of CTHRC1 in different types of human cancers in TIMER database. **B** Volcano plot of differentially expressed genes between tumor and normal tissue in HNSCC. **C** Expression of CTHRC1 in HNSCC from GEPIA database. **D** Expression of CTHRC1 in HNSCC、dysplasia、and normal tissue in GSE30784. **E** Expression of CTHRC1 in tumor and normal tissue in HPA database. (Normal: Female, age 59 ID4109. Tumor: Female, age 55, ID4420)

TIMER indicated a significant increase in the expression of CTHRC1 across various tumors, particularly in digestive tract tumors (Fig. 1A). For HNSCC, 1815 genes were up-regulated, while 1947 genes were down-regulated. CTHRC1 ranked 89th in the up-regulated genes. Compared with the adjacent normal tissues, the expression of CTHRC1 was significantly increased in the tumor tissues with a LogFC value of 3.5 (Fig. 1B). Comparable results were observed in the GEPIA database and HPA database (Fig. 1C and E). The results were also confirmed in the GEO database (Fig. 1D).

### Higher expression of CTHRC1 was found in advanced stage of HNSCC patients and related to poor prognosis

**Fig. 2:**
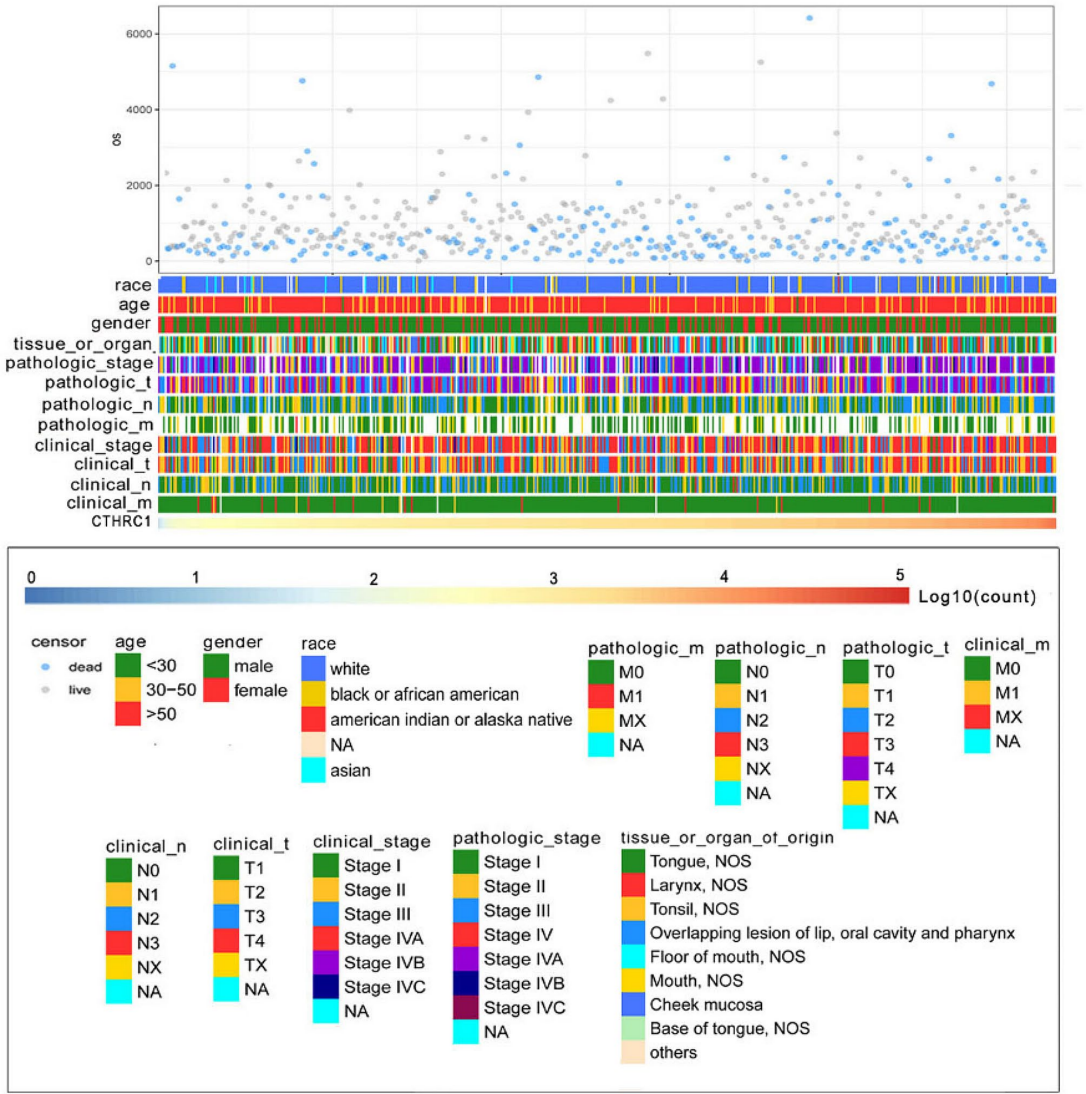
Landscape of CTHRC1-related clinicopathological features in HNSCC from TCGA

**Fig. 3:**
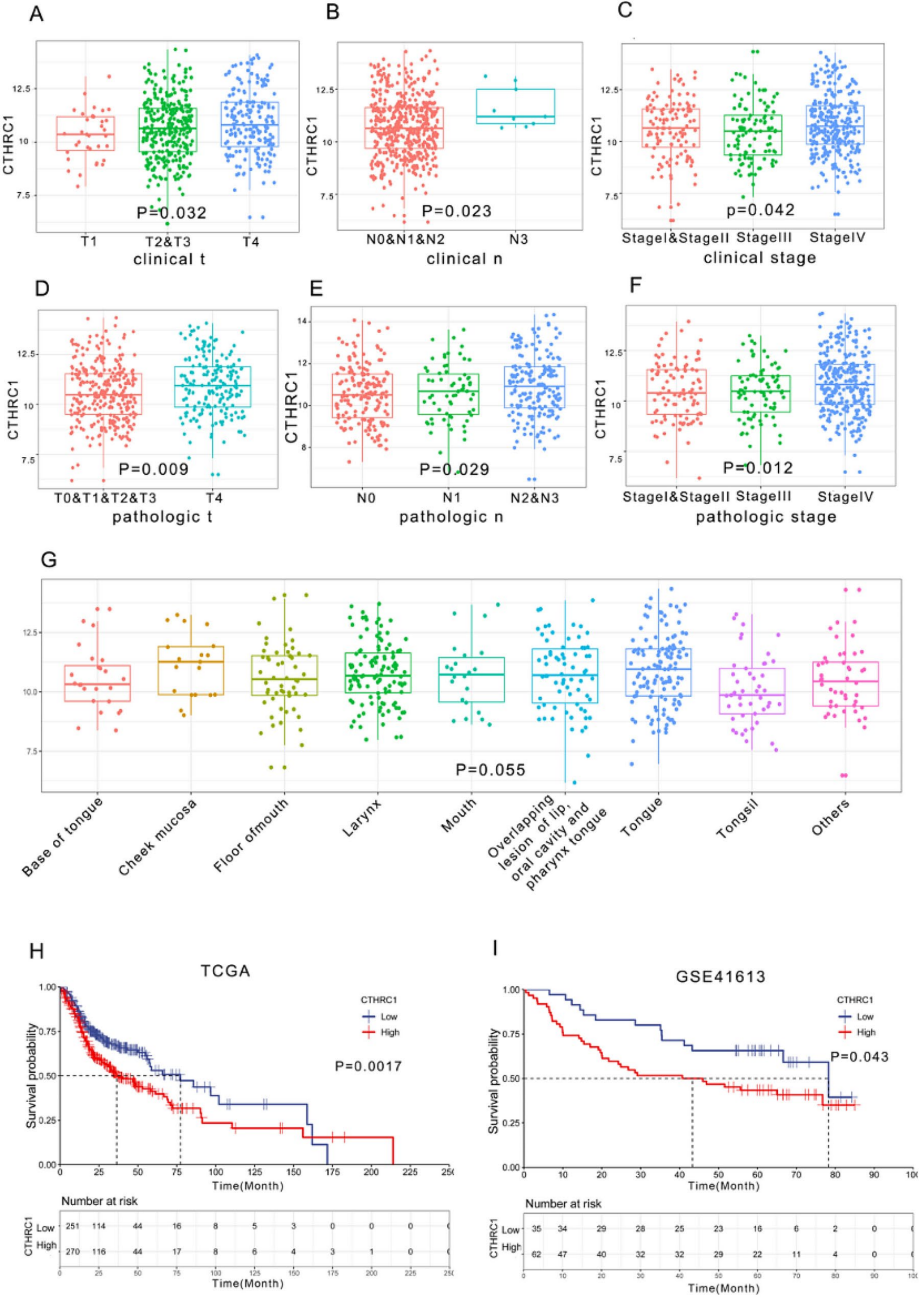
High expression of CTHRC1 is observed in late stage of HNSCC and predicts a poor prognosis. **A-F** CTHRC1 expression across different stages in HNSCC. **G** CTHRC1 expression across different organs or tissues. **H-I** Prognostic impact of CTHRC1: Kaplan-Meier survival curves in TCGA and GSE41613. Statistical significance was determined using either an unpaired t-test or one-way analysis of variance (ANOVA)

The heatmap illustrates the distribution of clinicopathological features and survival status among patients with increasing CTHRC1 expression in TCGA (Fig. 2). No significant differences were observed among different age groups, genders, M stages and races. CTHRC1 expression was significantly upregulated in the clinical T4 and N3 group (Fig. 3A and B). Further analysis revealed remarkable variation in mRNA expression levels of the CTHRC1 gene among patients with HNSCC at different clinical stages. The expression of CTHRC1 was significantly higher in clinical stage IV, whereas there was no significant difference among stages I, II, and III (Fig. 3C). Regarding pathological staging, the expression levels of the CTHRC1 gene were higher in the T4 and N2&N3 group (Fig. 3D and E). Furthermore, higher expression of CTHRC1 was observed in pathological staging IV (Fig. 3F). Regarding tissue or organ origin, the CTHRC1 expression level was highest in the overlapping lesion of the lip, oral cavity, and pharynx, and lowest in tonsils; however, there was no statistical significance (Fig. 3G).

The Kaplan-Meier Plotter was employed for survival analysis. To assess the OS of patients, 270 cases were classified into the high expression group, and 251 cases were assigned to the low expression group using maximally selected rank statistics in TCGA. Kaplan-Meier curves revealed that high CTHRC1 expression indicated a higher risk of poor overall survival (*P* = 0.0017). The median overall survival of the high and low CTHRC1 expression groups was 34.6 and 77.3 months, respectively (Fig. 3H). The above results were validated in the GEO database (GSE41613). The high group exhibited worse survival than the low group (*P* = 0.043). The median survival of the high group was 43.3, and for the low group, it was 78.3(Fig. 3I).

### Go, KEGG and GSVA analysis

**Fig. 4:**
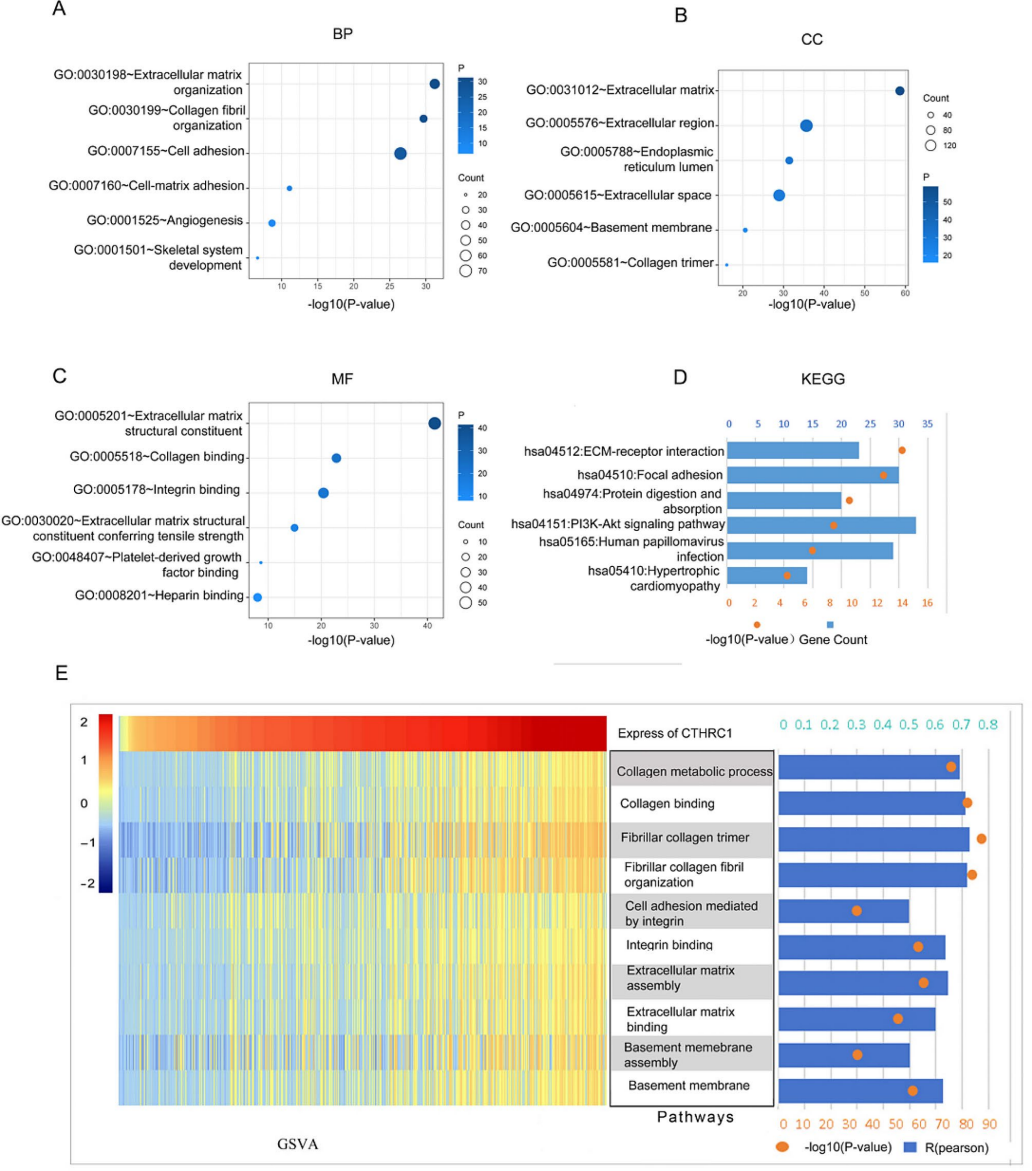
CTHRC1 is closely associated with ECM-related process. **A-C**, Biological processes (BP), cellular components (CC), and molecular functions (MF) associated with CTHRC1 in TCGA Database. **D** Kyoto encyclopedia of genes and genomes (KEGG) pathway analysis of CTHRC1 in TCGA. **E** Enrichment scores analysis between CTHRC1 expression and ECM-related pathways by GSVA

The top 500 co-expression genes were used for GO and KEGG analysis. The GO analysis revealed that these genes were enriched in processes such as extracellular matrix organization (BP: GO: 0030198), collagen fibril organization (BP: GO: 0030199), extracellular matrix (CC: GO:0031012), extracellular region (CC: GO: 0005576), (MF: GO: 0005201) and collagen binding (MF: GO: 0005518) (Fig. 4A and C). Additionally, the KEGG pathway analysis revealed pathways in which these genes were enriched, including ECM-receptor interaction, focal adhesion, protein digestion and absorption, PI3K-Akt signaling pathway, Human papillomavirus infection, and hypertrophic cardiomyopathy (Fig. 4D). Furthermore, GSVA analysis was conducted to reveal the physiological processes in which CTHRC1 might be involved. The results indicated that CTHRC1 might be associated with the formation and organization of collagen and fibril, extracellular matrix, and basement membrane (Fig. 4E).

### PPI network construction

**Fig. 5:**
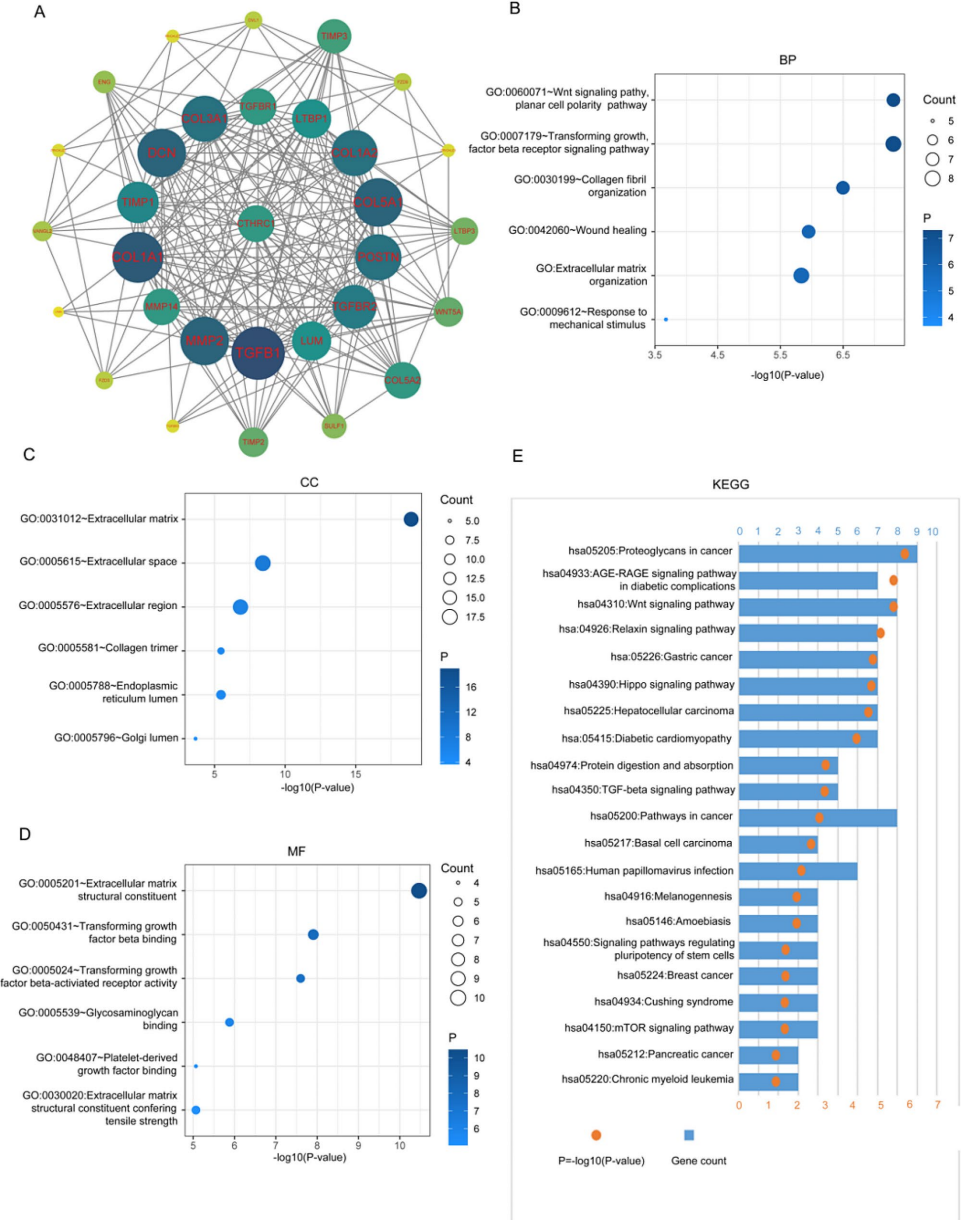
The protein-protein interaction network for CTHRC1. **A**. Protein-protein interaction network constructed by STRING. **B-D** Biological processes (BP), cellular components (CC), molecular functions (MF) and Kyoto encyclopedia of genes and genomes (KEGG) pathway analysis of genes encoding these proteins

The protein-protein interaction network for CTHRC1 was constructed using the STRING database. Thirty genes were found to be closely related to CTHRC1, many of which are involved in cancer biology, including TGFBR1, TGFBR2, TGFB1, MMP2, MMP14, TIMP1, TIM3, WNT5A, ENG, COL1A1, COL1A2, and POSTN (Fig. 5A). To explore the biological role and pathways of the related genes, GO and KEGG were used. Similar to CTHRC1, genes encoding these proteins were closely related to the formation and organization of collagen and fibril, extracellular matrix, and basement membrane. In addition, the result of KEGG indicated that many cancers have been linked to these genes, and many classical tumor-related pathways might be involved (Fig. 5B-E).

### Relationship between CTHRC1 and immune checkpoint genes

**Fig. 6:**
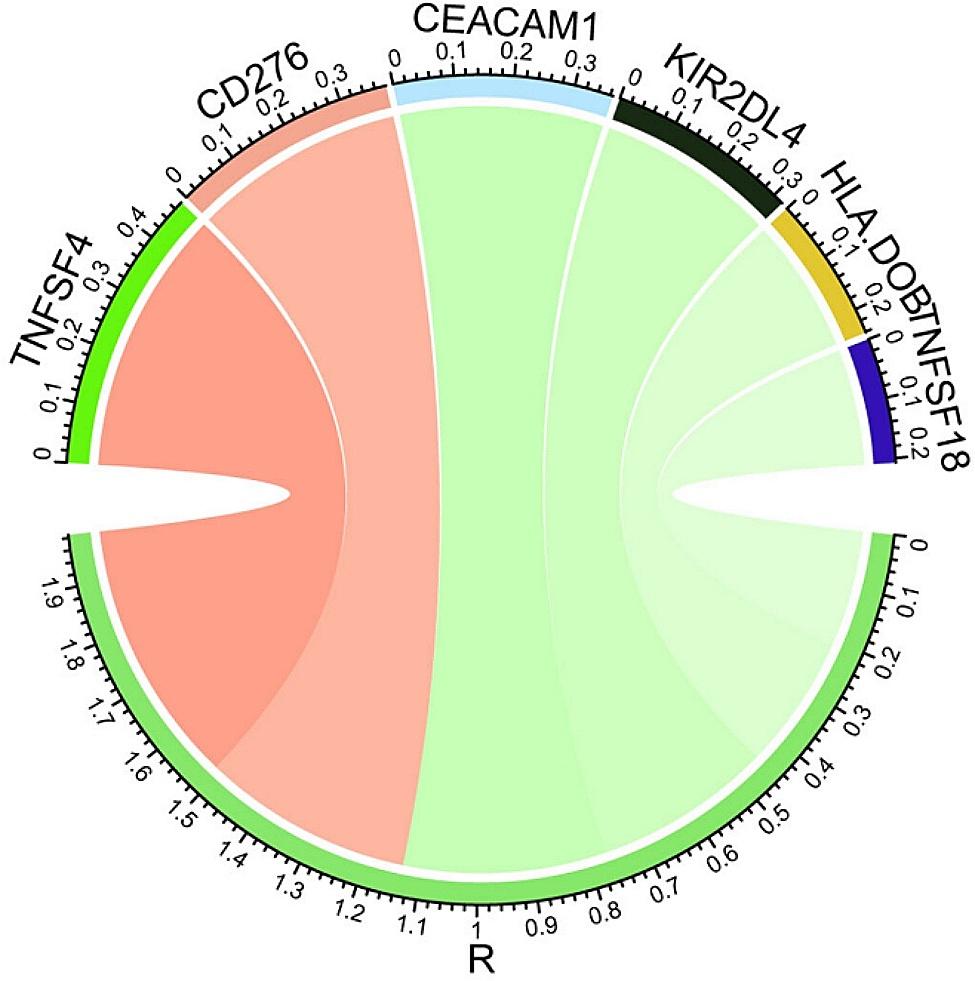
Pearson correlation between CTHRC1 and immune checkpoints. The width of the band represented the R-value. Red: positive correlation, Green: negative correlation

We explored the relationship between the expression of CTHRC1 and immune checkpoint genes using Pearson correlation analysis. Based on the previous literature, 79 immune checkpoint genes were selected. Finally, 19 genes were confirmed to be positively correlated with CTHRC1 expression in HNSCC (*R* > 0, *P* < 0.05), while 24 genes were negatively correlated (*R* < 0, *P* < 0.05) (Table 1). The top 6 checkpoints were TNFSF4 (*R* = 0.474, *P* = 1.25 × 10^− 30^), CD276 (*R* = 0.379, *P* = 2.94 × 10^− 19^), CEACAM1 (*R*=-0.365, *P* = 7.36 × 10^− 18^), KIR2DL4 (*R*=-0.365, *P* = 7.36 × 10^− 18^), HLA-DOB (*R*=-0.241, *P* = 2.32 × 10^− 8^), and TNFSF18 (*R*=-0.208, *P* = 1.70 × 10^− 6^) (Fig. 6).

**Table 1:** Pearson correlation between CTHRC1 and immune checkpoints

Checkpoints	Negative		Checkpoints	Positive	
	*R*	*P*		*R*	*P*
CEACAM1	-0.364637	7.36E-18	TNFSF4	0.474232	1.25E-30
KIR2DL4	-0.318461	9.09E-14	CD276	0.378833	2.94E-19
HLA-DOB	-0.241447	2.32E-08	HAVCR2	0.181044	3.17E-05
TNFSF18	-0.207682	1.70E-06	TNFRSF9	0.178054	4.29E-05
CD47	-0.198672	4.79E-06	SIRPA	0.176806	4.87E-05
CD160	-0.192733	9.22E-06	TDO2	0.174897	5.89E-05
KIR2DL3	-0.188442	1.46E-05	CD86	0.165919	1.40E-04
IDO1	-0.184338	2.26E-05	BTN2A1	0.158072	2.88E-04
PDCD1	-0.182631	2.69E-05	VTCN1	0.148297	6.77E-04
LAG3	-0.156677	3.27E-04	HLA-DQA1	0.130611	2.79E-03
KIR3DL2	-0.156003	3.47E-04	HLA-DPB1	0.129081	3.13E-03
ICOSLG	-0.146803	7.67E-04	CD70	0.112018	1.04E-02
KIR3DL3	-0.145691	8.42E-04	CD40	0.101358	2.05E-02
KIR3DL1	-0.145139	8.81E-04	HLA-DQB1	0.095042	2.99E-02
CD274	-0.132283	2.46E-03	HLA-DPA1	0.093850	3.20E-02
KIR2DL1	-0.127336	3.57E-03	PVR	0.093065	3.35E-02
CD226	-0.124656	4.34E-03	HLA-DRA	0.091449	3.67E-02
HLA-E	-0.122916	4.92E-03	HLA-DOA	0.087636	4.54E-02
CD40LG	-0.104757	1.67E-02	HLA-DMB	0.087402	4.59E-02
BTLA	-0.104137	1.73E-02			
TNFSF9	-0.103537	1.80E-02			
BTNL3	-0.092161	3.53E-02			
CD27	-0.091573	3.65E-02			
CD96	-0.090850	3.80E-02			

### CTHRC1 expression and immune cell infiltration

**Fig. 7:**
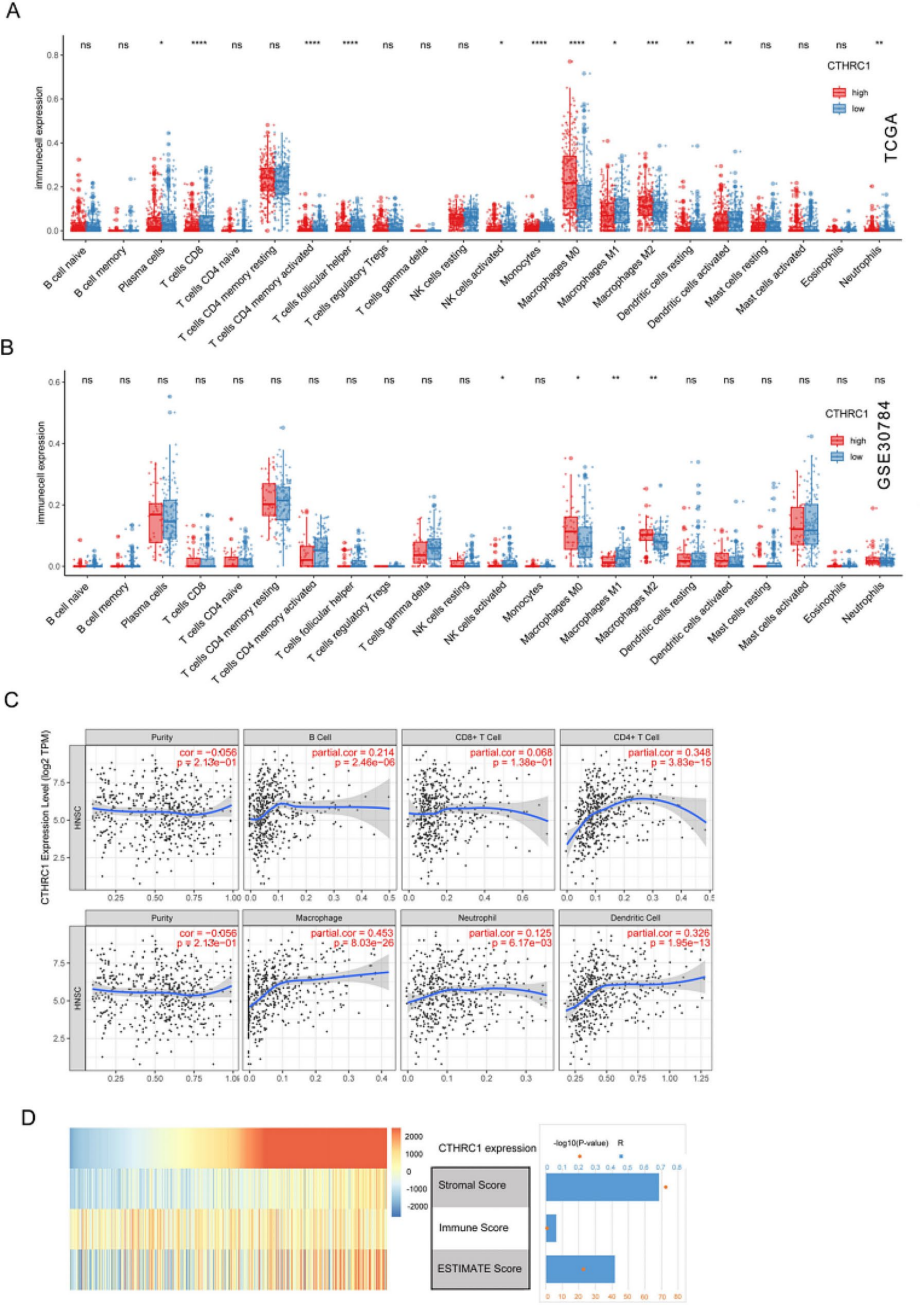
Immune infiltration analysis of CTHRC1 expression in HNSCC. **A** Infiltration of different immune cells in CTHRC1 high expression group and low expression group analyzed by CIBERSORT in TCGA. **B** Infiltration of different immune cells in CTHRC1 high expression group and low expression group in GSE30784. **C** Correlation of CTHRC1 expression with immune infiltration levels in TIMER. **D** Pearson correlation between CTHRC1 and immune score using ESTIMATE

In this study, we conducted an analysis of immune cell infiltration features using CIBERSORT, TIMER, and ESTIMATE. The CIBERSORT results revealed that 12 out of the 22 types of immune cell types were differentially expressed between CTHRC1 high and low expression groups in TCGA, including plasma cells, T cells CD8, T cells CD4 memory activated, T cells follicular helper, NK cells activated, Monocytes, Macrophages (M0, M1, M2), Dendritic cells resting, Dendritic activated, and Neutrophils (Fig. 7A). In GSE30784, only 4 types of immune cells were discovered (NK cells activited, Macrophages M0, Macrophages M1 and Macrophages M2) (Fig. 7B). Similar findings were also observed, with the exception of CD8 cells, which showed no significant association with CTHRC1 expression in the TIMER database (Fig. 7C). ESTIMATE indicated a correlation between Stromal Score and ESTIMATE score with CTHRC1 expression. However, no correlations were found between CTHRC1 expression and Immune Score (Fig. 7D). Since the ESTIMATE Score is negatively correlated with tumor purity, we infer that tumor purity is negatively correlated with CTHRC1 expression. This finding contradicted the conclusions drawn in the TIMER database, which showed no correlations between CTHRC1 expression and tumor purity.

## Discussion

HNSCC is one of the most prevalent cancers globally, posing a significant threat to human health. The management of HNSCC varies depending on the stage of the disease, anatomical location, and surgical accessibility. In the early stages (stage I and stage II), smaller tumors may undergo extensive resection directly. Intriguingly, diode lasers have gradually been adopted in surgical treatments, enhancing operational efficiency and yielding exceptional aesthetic and functional outcomes [40, 41]. In advanced stages (stage III and stage IV), surgery remains the primary method, with the removal of the primary lesion accompanied by neck lymph node dissection. Postoperative radiotherapy or chemotherapy may be administered subsequently [42, 43]. In recent years, targeted therapies exemplified by EGFR inhibitors, and immunotherapies, such as pembrolizumab hold immense potential in the treatment of advanced HNSCC [26, 44].

The aberrant expression of CTHRC1 has been documented in various cancers, including breast cancer, stomach adenocarcinoma, non-small cell lung cancer, and colon adenocarcinoma [19–22]. The understanding of the expression pattern of CTHRC1 in tumors will help us to understand the pathogenesis of tumors. Experimental research for HNSCC has indicated that N-Glycosylation collaborates with canonical Wnt pathway to induce CTHRC1 and drive HNSCC cell migration [45]. The high expression of CTHRC1 may be associated with HNSCC progression and poor prognosis, helping identify high risk individuals. Detecting CTHRC1 aids in the early detection and diagnosis of HNSCC, improving treatment outcomes through noninvasive methods. Further research on CTHRC1 will help develop new diagnostic and therapeutic strategies. In the present study, we found that CTHRC1 expression was significantly higher in tumor tissues consistent with lee’s study in which the HNSCC samples showed a 12.3fold higher expression than normal samples [23]. This suggests that elevated CTHRC1 expression may be associated with a higher risk of HNSCC, and CTHRC1 can serve as a tumor marker to predict the risk of HNSCC occurrence.

Timely diagnosis and early surgery in the initial stages of the tumor are crucial factors affecting the prognosis of HNSCC. It has been asserted that delayed diagnosis and treatment of patients with HNSCC worsen prognosis outcomes and increase undesirable morbidity and mortality in cancer patients [46, 47]. In this study, we observed that CTHRC1 expression tends to be up-regulated in advanced stages of head and neck squamous cell carcinoma. Additionally, the OS of the high CTHRC1 expression group is significantly worse than that of the low expression group. This trend has been confirmed in both the TCGA and GSE41613 datasets. Therefore, high expression of CTHRC1 may predict poor prognosis of patients which is consistent with findings of Lee’s study [23]. The prognostic value of CTHRC1 has also been found in other tumors, such as colon adenocarcinoma, gastric cancer, breast cancer and kidney renal clear cell carcinoma [20, 48, 49]. Our results indicates that CTHRC1 can also serve as a marker to predict the stage and prognosis of HNSCC.

Prior studies have indicated that TME is implicated in various pathological processes of tumor, including tumor growth, metastasis, and invasiveness [29, 30]. Being a dynamic interconnected mesh of macromolecules, ECM stands out as the most abundant component in TME, offering structural support and regulating cellular behavior through mechanical and biochemical cues [50]. It regulates various cellular processes, including proliferation, differentiation, migration, invasion, and survival in cancer [50, 51]. In this study, genes related to CTHRC1 expression in HNSCC were identified using the TCGA database. POSTN, SPARC, PLPP4, GLT8D2, and THY1 were identified as the five genes most closely expressed to CTHRC1, suggesting a robust link between CTHRC1 and the relevant functions of the ECM. Subsequently, we explored the physiological function and related pathways using GO, KEGG, and GSVA analyses. The results indicated that CTHRC1 and its coexpressed genes are enriched in ECM-related processes, such as extracellular matrix organization, collagen fibril organization, cell adhesion, and cell matrix adhesion. In previous studies, Zhao found that CTHRC1 was related to several pathways in gastric cancer, including extracellular matrix organization and vascular development, through enrichment analysis [20]. Also, a similar result was found in meng’s study of colon cancer [48]. Our results were consistent with their studies.

The protein-protein interaction network consists of proteins that interact to participate in various life processes, including biological signal transmission, gene expression regulation, energy and substance metabolism, and cell cycle regulation [52]. In this study, a protein-protein network was constructed, revealing 30 related proteins, including COL3A1, TGFBR1, LTBP1, COL1A2, COL5A1, and POSTN. KEGG analysis showed that, in addition to ECM-related physiological processes, these proteins were also implicated in the initiation and progression of various cancers, as demonstrated in other studies. Zhao indicate that ECM-related genes correlate with immune cells, overall survival, and recurrence of bladder cancer [53]. Also, Keerthi proved that matrisome genes were seen to affect survival across cancers by a pan-cancer analysis [54]. Furthermore, these proteins were involved in several classical signaling pathways that have been proven to play important roles in tumors, such as the Wnt signaling pathway, Hippo signaling pathway, and mTOR signaling pathway [55].

Head and neck cancer inherently exhibits immunosuppression, prompting research into the role of the immune landscape in HNSCC [56]. The advent of immune checkpoint inhibitors represents a noteworthy advancement in oncological therapy. Immune checkpoint blockade (ICB) therapies targeting PD1 and PDL1 have been approved to treat various malignancies, yielding some therapeutic effects. Despite these advancements, only a small percentage of HNSCC patients respond to ICB, with studies indicating a benefit for only 10–20% of HNSCC patients [26, 27]. Therefore, there is an urgent need for the identification of the most suitable treatment regimens and the reduction of immunosuppression in non-responding patients with head and neck cancer. Immune checkpoint agents exhibit antitumor properties by reversing tumor immunosuppressive effects. The study also investigated the correlation between the signature and the levels of various immune checkpoint proteins. Patients with higher CTHRC1 expression exhibited elevated expressions of TNFSF4 and CD276. TNFSF4, a cytokine that promotes the activation and proliferation of T cells, is a well-known immune checkpoint. A previous study demonstrated that TNFSF4 could facilitate chemoresistance in lung adenocarcinoma by inhibiting the apoptosis of tumor cells [57]. The cell surface molecule CD276 serves as an immune checkpoint antigen. Elevated expression of CD276 on tumors contributes to the suppression of anti-tumor T-cell responses and correlates with a poor prognosis [58]. Consequently, the results of this study suggest that TNFSF4 and CD276 may be novel targets for HNSCC immunotherapy.

In addition to stromal cells, immune cells constitute crucial components of the TME and play a pivotal role in the onset and progression of tumors. Studies have indicated that the immune infiltration status of tumors is a crucial factor influencing tumor growth and prognosis [59]. In our study, we assessed the tumor’s immune infiltration status using ESTIMATE, TIMER, and CIBERSORT. ESTIMATE calculates immune and stroma scores by analyzing gene expression signatures specific to immune and stroma cells to predict infiltration by non-tumor cells. The results indicated no correlation between CTHRC1 and the immune score, but it can influence the infiltration status of non-tumor cells by impacting the stromal score. While not influencing the overall immune score, CIBERSORT demonstrated a significant impact of CTHRC1 on the composition of immune cells in tumors, particularly macrophages. Macrophages can polarize into either M1-like or M2-like macrophages. M1-like macrophages are pro-inflammatory and play a role in host defense by producing cytokines like IL-12 and TNF-α, and promoting Th1 responses. In contrast, M2-like macrophages are anti-inflammatory, involved in tissue repair, and secrete cytokines like IL-10 and TGF-β, promoting Th2 responses [60]. Macrophages in the tumor microenvironment are termed tumor-associated macrophages (TAMs), representing a distinctive phenotype of M2-like macrophages [61, 62]. It has been demonstrated that TAMs within the tumor microenvironment promote tumor growth, angiogenesis, and metastasis by secreting pro-tumorigenic factors and suppressing antitumor immune responses across various cancer types [63]. The study revealed a significant increase in M2 macrophages and a corresponding decrease in M1 macrophages in the CTHRC1 high-expression group. This alteration implies that CTHRC1 may be linked to the imbalance in macrophage proportion, a factor deemed crucial in tumor development, immune evasion, and subsequent metastasis and drug resistance, which was also proved in other cancers [22, 49, 61, 64]. Taken together, these findings suggest that CTHRC1 might serve as an indicator of HNSCC prognosis by influencing immune infiltration status, particularly in relation to M2 macrophages. Immunotherapy targeting macrophage polarization may offer potential benefits for improving the treatment outcome of HNSCC patients, with CTHRC1 emerging as a promising target. However, further studies are needed to confirm the causal relationship between them.

In this study, we investigated the relationship between CTHRC1 and HNSCC, aiming to elucidate the underlying mechanisms. Nevertheless, this study has certain limitations. All the data utilized in this study were obtained from online databases, lacking experimental validation and may have biases such as measurement errors and data missing. Also, we did not draw a clear boundary to distinguish high expression and low expression. Additionally, some inconsistent results, such as the disparity between TIMER and ESTIMATE in the assessment of tumor purity, remain unexplained. Therefore, additional analyses and experiments, encompassing in vitro and in vivo validations, are necessary to validate our findings.

## Conclusions

High expression of CTHRC1 predicts poor prognosis and is associated with immune infiltration in HNSCC, confirming its utility as a tumor marker for HNSCC.

## Data Availability

Data that support the findings of this study can be obtained from the public database TCGA-HNSC and GEO database (GSE30784 and GSE41613).

## Abbreviations

BP: Biological process
CC: Cellular component
CIBERSORT: Cell-type Identification By Estimating Relative Subsets Of RNA Transcripts
CTHRC1: Collagen triple helix repeat containing-1
DAVID: The database for annotation, visualization, and integrated discovery
DEGs: Differentially expressed genes
ECM: Extracellular matrix
ESTIMATE: Estimation of Stromal and Immune Cells in Malignant Tumour Tissues using Expression Data
GEO: Gene expression omnibus
GEPIA: Gene Expression Profiling Interactive Analysis
GO: Gene ontology
GSVA: Gene set variation analysis
HNSCC: Head and neck squamous cell carcinoma
HPA: Human protein atlas database
ICB: Immune checkpoint blockade
KEGG: Kyoto encyclopedia of genes and genomes
MF: Molecular function
OS: Overall survival
PPI: Protein-protein interaction
STRING: The search tool for the retrieval of interacting genes
TAMs: Tumor-associated macrophages
TCGA: The cancer genome atlas
TIMER: Tumor Immune Estimation Resource
TME: Tumor microenvironment
